# ServAR: An augmented reality tool to guide the serving of food

**DOI:** 10.1186/s12966-017-0516-9

**Published:** 2017-05-12

**Authors:** Megan E. Rollo, Tamara Bucher, Shamus P. Smith, Clare E. Collins

**Affiliations:** 1 School of Health Sciences, Priority Research Centre for Physical Activity & Nutrition, ATC Building, University of Newcastle, Callaghan, NSW 2308 Australia; 2 Institute for Environmental Decisions, ETH Zurich, Zurich, Switzerland; 3 School of Electrical Engineering and Computing, University of Newcastle, Callaghan, Australia

**Keywords:** Augmented reality, Estimation error, mHealth, Nutrition, Portion control

## Abstract

**Background:**

Accurate estimation of food portion size is a difficult task. Visual cues are important mediators of portion size and therefore technology-based aids may assist consumers when serving and estimating food portions. The current study evaluated the usability and impact on estimation error of standard food servings of a novel augmented reality food serving aid, ServAR.

**Methods:**

Participants were randomised into one of three groups: 1) no information/aid (control); 2) verbal information on standard serving sizes; or 3) ServAR, an aid which overlayed virtual food servings over a plate using a tablet computer. Participants were asked to estimate the standard serving sizes of nine foods (broccoli, carrots, cauliflower, green beans, kidney beans, potato, pasta, rice, and sweetcorn) using validated food replicas. Wilcoxon signed-rank tests compared median served weights of each food to reference standard serving size weights. Percentage error was used to compare the estimation of serving size accuracy between the three groups. All participants also performed a usability test using the ServAR tool to guide the serving of one randomly selected food.

**Results:**

Ninety adults (78.9% female; a mean (95%CI) age 25.8 (24.9–26.7) years; BMI 24.2 (23.2–25.2) kg/m^2^) completed the study. The median servings were significantly different to the reference portions for five foods in the ServAR group, compared to eight foods in the information only group and seven foods for the control group. The cumulative proportion of total estimations per group within ±10%, ±25% and ±50% of the reference portion was greater for those using ServAR (30.7, 65.2 and 90.7%; respectively), compared to the information only group (19.6, 47.4 and 77.4%) and control group (10.0, 33.7 and 68.9%). Participants generally found the ServAR tool easy to use and agreed that it showed potential to support optimal portion size selection. However, some refinements to the ServAR tool are required to improve the user experience.

**Conclusions:**

Use of the augmented reality tool improved accuracy and consistency of estimating standard serve sizes compared to the information only and control conditions. ServAR demonstrates potential as a practical tool to guide the serving of food. Further evaluation across a broad range of foods, portion sizes and settings is warranted.

## Background

The portion size of many food and drinks continue to increase. Longitudinal data on dietary intakes indicates that there has been a sustained increase in the portion size of most foods in the USA since the 1970s [1, 2]. In other countries, such as Australia [3] and Ireland [4], temporal increases in portions size have also been reported for some foods. Factors identified as contributing to the consumption of larger portions include perceptions of ‘value for money’, increased sizes of pre-packaged foods, drinks, serving vessels and tableware, sustained exposure to larger portions through the food environment, and a lack of awareness or understanding of recommended serving sizes [5–7].

Offering larger food portion sizes is associated with an increase in energy intake. Multiple laboratory-based studies, in addition to those in free-living settings, have shown that the presentation of larger portion sizes result in an increase in the food amount and energy consumed [5–8]. Although a definitive link between larger portion sizes and obesity remains to be established [6], a recent meta-analysis of 58 studies demonstrated a small to moderate effect of exposure to larger food portions and packages, and tableware is associated with increased energy intake [5]. The review by Hollands and colleagues [5] found strategies to reduce exposure to larger food portions can produce meaningful reductions in overall intake of 189 kcal per day (144 kcal to 228 kcal) [5].

Visual cues appear to be an important mediator of the portion size effect or the impact of larger portion sizes on intake. Characteristics of serving vessels, packaging and individual food unit size, along with the amount served act as a reference to the amount to be consumed (i.e. anchoring and adjustment norms), significantly influences portion size perception [9]. However, visual perception of portion size alone is considered unreliable due to biases triggered by the food itself (e.g. ‘healthy’ vs. ‘unhealthy’ foods) or the microenvironment (e.g. changes in food packaging dimensions and label information of food products) [10]. Therefore, intervention opportunities for portion control may include physical, economic, political and socio-cultural components of food environment, in addition to strategies targeting individuals [11].

At the individual level, self-regulation of both type and amount of food served and consumed is acknowledged as a key behavioural strategy in portion control interventions [12]. Within the context of the broader food environment, where exposure to large portion sizes is unavoidable, enabling individuals with self-regulation skills for portion control is essential [13]. While education to manage portion size is important, interventions have previously been shown to be effective only in the short-term [13]. Further, perceived barriers to using portion control strategies relate to a lack of suitable aids and the view that guidance on appropriate portion size selection is not needed [14]. Consequently, common aids are rarely used for portion control in everyday life [15]. In addition to common aids such measuring cups, food scales and using parts of the hand (e.g. finger, palm, fist), a number of household items suggested for portion control include sporting balls (e.g. tennis, golf and baseballs), card decks, dice and compact discs [16]. These aids are thought to be readily available or items that most individuals have been regularly exposed to outside of their food environment. However, despite being purported as ‘practical’, there is large variation in the putative volumes and the measured volumes of these household items, in particular across different food types [16], further impacting use.

Mobile devices, such as smartphones (e.g. Apple iPhone®, Samsung Galaxy®) and tablet computers (e.g. Apple iPad®), combine portability with features such as a camera and network connectivity, and are suitable for the delivery of nutrition education resources to guide portion control at the time of eating. These devices are now common with ownership of smartphones being highest among Australian adults (77%), followed by the United States (72%) [17]. Augmented reality (AR) is a type of technology that is well suited to delivery via mobile devices. AR involves the overlay of computer-generated or virtual content onto objects present in reality and aims to augment the user experience [18]. In the context of health behaviours, AR has been used extensively in the treatment of phobias using exposure-based therapies [19, 20]. A limited number of AR experiences to support individuals in the estimation of self-served portion sizes have been tested [21, 22]. To date, these experiences have been developed as standalone mobile applications, with a high level of user dexterity required to input information using touch gestures, resulting in some individuals finding the experience difficult [21, 22]. In an effort to improve the user experience and minimise the amount of interaction with the mobile touch screen, we used an alternative approach to develop ServAR, an AR tool to guide the serving of food for portion control. This study aimed to evaluate ServAR’s usability and impact on error associated with serving commonly consumed foods.

## Methods

### Selection of test foods

**Fig. 1:**
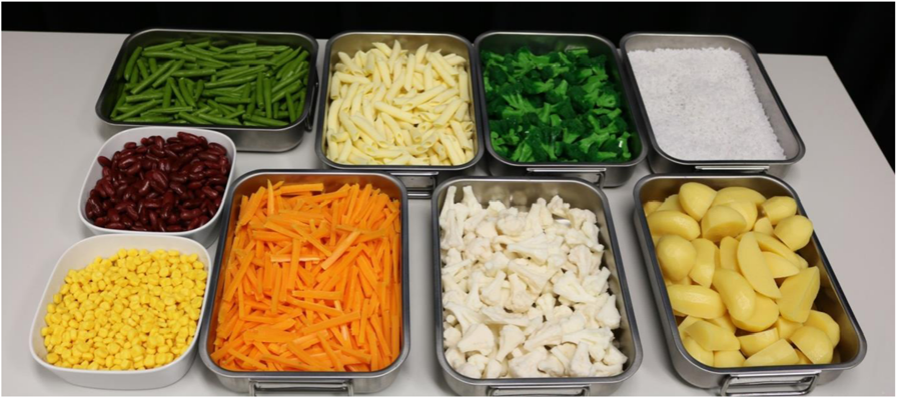
Replica foods used in experiment. Clockwise from the top left: green beans, pasta (*penne*), broccoli, rice, potatoes, cauliflower, carrots, sweetcorn, and kidney (*red*) beans

Based on guidelines for the development of portion size aids [23], test foods included in the current study were those that varied in portion size along a continuum and were not available in commercially standardized amounts (e.g. slice of bread). For the study, we used authentic food replicates (Doering GmbH, Germany), which are validated [24] and used for portion size and serving studies [25–28]. For the purpose of testing the effect of AR to guide the serving of food, nine test foods were selected: broccoli, carrots, cauliflower, green beans, kidney beans, pasta (penne), potatoes, rice, and sweet corn. Potatoes were cut into various sized pieces (Fig. 1). The remaining foods were amorphous in type or tend to not have a predefined shape and mound or take on the shape of the serving vessel in which they are served.

To test the impact of ServAR on serving accuracy for the purpose of this proof-of-concept study, a *reference serve* for each test food was set to replicate the standard serve sizes of the national food selection resource, the Australian Guide to Healthy Eating (AGHE) [29]. For all nine test foods, each reference serve was equal to half a standard measuring cup (125 mL) or equivalent to one AGHE standard serve size [29]. Within the AGHE kidney beans can be categorised as either a vegetable or meat alternative with different standard serving sizes (1/2 cup vs. 1 cup; respectively) [29]. For the purposes of the current study kidney beans were categorised as a vegetable. To determine the average weight of the reference serve for each test food, triplicate measures using a standard 1/2 household cup (loosely packed) were collected using the replica foods. A conversion factor was applied to convert the weight of the replica foods to their real food equivalent weight.

### Development of the ServAR tool

The ServAR tool consisted of an image overlay for each of the nine test foods in amounts depicting the AGHE standard serve size. Images were captured using a digital single reflex camera (Canon 5D Mark III - 22.3MP with a Canon EF 24-105 mm lens). The camera was fixed at an angle of 45° and mounted on a tripod. The diagonal distance between the plate and the camera was 89.5 cm. Two flashes (Canon 580EX @ 1/2 power) were mounted on a stand with a ‘shoot through’ umbrella and angled at 45° to plate on both sides of the camera. Foods were served onto a white dinner plate (29.5 cm diameter) using an Australian measuring cup (1/2 cup; loosely packed). A fiducial marker (9 cm × 5 cm card) was placed next to the plate and remained fixed throughout the collection of the images. Both the plate and marker were placed on a white cardboard background.

The virtual food objects were produced from images captured of the food portions and then modified using photo editing software. Initially basic corrections (e.g., exposure, highlights, shadows, contrast) were applied to all images using Adobe Photoshop Lightroom® 4 (Adobe Systems Software Ltd., Ireland). Photoshop® CS5 and CC2015 (Adobe Systems Software Ltd., Ireland) were then used to remove the background of each image leaving only the food served and the fiducial marker. An outline was applied to the fiducial marker in a contrasting colour and the fiducial marker then removed from the image. For the images of corn, green beans, kidney beans, carrots, broccoli and boiled potatoes the opacity of these images was changed to 50% (i.e., so that the virtual food objects were transparent). In addition, for white-coloured foods (i.e., pasta, rice, and cauliflower) a contrasting colour mask at 10–15% opacity also applied to the food within the image to add contrast against the plate. Images were cropped to optimise use in the AR platform.

**Fig. 2:**
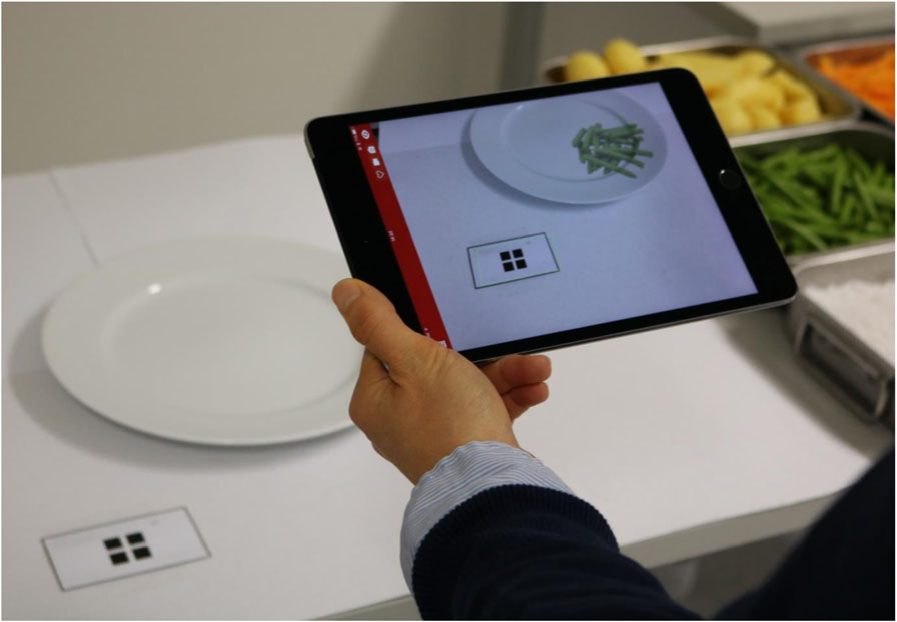
The ServAR tool. The tool comprised virtual objects consisting of the test food (e.g. green beans) in the reference portion size and an outline of the fiducial marker displayed on an iPad® Mini using the Zappar application. The iPad® Mini was fixed to a stand when used by the ServAR group during the main experimental study. Participants held the iPad® Mini during the usability activity in a manner similar to the demonstration in the figure

To create the ServAR tool, the virtual food objects were incorporated into the web-based AR platform, ZapWorks (Zappar Ltd., United Kingdom). The platform enables the upload of multimedia content to create an AR experience that allows the content to be overlayed virtually when viewed through the accompanying mobile device application, Zappar. The application uses the camera of the mobile device to scan a code which triggers the virtual object(s) to appear on the device’s screen and be overlayed on the content present in reality. In the current study, an iPad® Mini (Apple Inc., Cupertino, USA) was used to view the AR experience, with each virtual food serving (along with an outline of the fiducial marker) being displayed on the iPad® Mini screen (Fig. 2).

### ServAR study

As the intention of the study was to pilot the ServAR tool it was determined a priori to recruit 30 participants per group. Ninety participants aged 18–35 years and not trained in or currently studying nutrition and dietetics were recruited to the experimental laboratory on campus to participate in a study about food choice. Recruitment occurred over ~6 months via the University of Newcastle and affiliated networks using various strategies, including print advertisements around the campus, course microsites and social media posts. The study was approved by the Human Research Ethics Committee of the University of Newcastle (H-2015-0306). All participants provided written informed consent and received a beverage voucher for participation. Students undertaking eligible courses were provided with bonus credit for participation as part of a research awareness initiative for selected programs.

There were three parts to the study: Part 1, an experimental component; Part 2, a usability component; and Part 3, a survey.

### Part 1: Experimental procedure

Participants were randomly assigned into one of three experimental groups: 1) Control; 2) Standard information only; or 3) ServAR tool. Participants in each group received one of the following three serving instructions. Participants in the control group were asked to serve an amount that they thought was a standard serve of each food without receiving any information or use of an aid. Participants in the standard information group were informed verbally that a standard serve was half a cup and then asked to serve a standard serve of each food. Those in the ServAR group were asked to serve a standard serve of each food using the ServAR tool as an estimation aid. An iPad Mini® was used for the ServAR tool and the device fixed to a stand to ensure the device remained stationary during the estimations.

For Part 1, each participant was presented with the nine replica foods. The foods were set up in a buffet style and the order in which the foods were presented was randomised for each participant. A tray of each food item was taken from the buffet and presented to the participant individually. Participants were then asked to serve a standard serving size of each food onto a plate (29.5 cm diameter). The quantity of each food served onto the plate by the participant was discreetly weighed by a research assistant using a digital scale before the next food was presented. This process was repeated until all nine foods had been served by the participant.

### Part 2: Usability activity

After the experimental Part 1, each participant was presented with one additional, randomly selected food from the nine previous food items in the usability test in Part 2. Here, all participants were instructed on how to use the ServAR tool to guide them when serving a portion of each food. Short instructions on how to hold the iPad® Mini and on how to align the marker on the table with the virtual marker were provided to participants (Fig. 2). Those who had difficulties aligning the markers were told that they could move freely and step towards or away from the table and/or hold the iPad® Mini at an approximate 45° angle to the table to facilitate alignment.

### Part 3: Survey

After completion of the serving tasks, participants were required to complete a survey (Qualtrics, LLC, Utah, USA) on a laptop. The survey assessed participant’s knowledge about the AGHE recommendations, their familiarity with the use of measurement aids, their smartphone use and dietary self-monitoring. Demographic information (e.g. age, height, weight, country of birth) on participants was also collected. All participants were asked to indicate their level of agreement using a six point scale (‘1’ = strongly agree to ‘6’ = strongly disagree) for nine questions relating to the usability of the ServAR tool. These questions evaluated three components of the ServAR tool, consisting of the ease of use of current features, perceived potential of the tool in everyday life, and any additional features (Table 3).

### Statistics

Data were analysed using IBM SPSS Statistics, Version 22 (SPSS. Inc., Chicago, IL, USA). Data are summarised as means and 95% confidence intervals (for normally distributed) or median and interquartile range (non-parametric data). Participant characteristics were compared between groups using ANOVA and Chi-square tests. Significance was set at *P* < .05. The weight of each participant’s serving of the replica food was converted into its real food equivalent using a conversion factor. The weight of the real food equivalent was used for all bivariate tests. Error was calculated in grams as the difference between the actual amount served and reference serving size. For each group, Wilcoxon signed-rank tests compared median amount served to reference serve for each food. The Bonferroni correction was applied to adjust for multiple comparisons with the corrected significance level of *P* < .006 used. In addition, percentage error was calculated (([served weight-reference serve weight]/reference serve weight)*100) to assess the level of estimation accuracy between the three groups. Box plots were used to visually inspect the distribution of estimation error by food across the three experimental conditions. Usability scores relating to ServAR tool were compared between groups using ANOVA.

## Results

### Participants

Table 1 summarises the characteristics of the 90 adults who were randomised into three groups and completed the study. Participants were predominantly female (78.1%), born in Australia (66.7%) and owned a smartphone (97.8%), with a mean (95%CI) age of 25.8 (24.9–26.7) years and a BMI of 24.2 (23.2–25.2) kg/m^2^. No significant differences in participant characteristics were found between groups. Most (56.7%) participants used measuring cups at home at least ‘several times per month’ or more frequently, compared to 53.3% reporting as ‘never’ using a scale at home to measure food. Overall, 52.2% had not heard of the AGHE serve sizes.

**Table 1:** Characteristics of participants

		*N* (%) or *Mean (95% CI)*							
		Condition							
		Control (*N* = 30)		Information only (*N* = 30)		ServAR tool (*N* = 30)		Total (*N* = 90)	
Age; *years*		26.5	(24.9 – 28.0)	25.6	(23.7 – 24.4)	25.4	(23.9 – 26.9)	25.8	(24.9 – 26.7)
BMI^a^; *kg/m* ^*2*^		24.5	(22.6 – 26.4)	24.2	(22.7 – 25.7)	23.9	(21.9 – 25.9)	24.2	(23.2 – 25.2)
Gender	Female	25	(83.3)	23	(76.7)	23	(76.7)	71	(78.9)
	Male	5	(16.7)	7	(23.3)	7	(23.3)	19	(21.1)
Country of birth	Australia	22	(73.3)	21	(70.0)	17	(56.7)	60	(66.7)
	Other	8	(26.7)	9	(30.0)	13	(43.3)	30	(33.3)
Do you currently monitor or track the amounts or portions of food you eat?	No	26	(86.7)	23	(76.7)	23	(76.7)	72	(80.0)
	Yes	4	(13.3)	7	(23.3)	7	(23.3)	18	(20.0)
Do you have a smartphone?	No	0	(0.0)	0	(0.0)	2	(6.7)	2	(2.2)
	Yes	30	(100.0)	30	(100.0)	28	(93.3)	88	(97.8)
How often do you use the following measurement aids at home:-									
Measuring cups^c^	Daily or several times per week	10	(33.3)	9	(30.0)	13	(43.3)	32	(35.6)
	Several times per month	12	(40.0)	13	(43.3)	7	(23.3)	32	(35.6)
	Once per month or less or never	8	(26.7)	8	(26.7)	10	(33.3)	26	(28.9)
Scales^d^	Daily or several times per week	6	(20.0)	3	(10.0)	5	(16.7)	14	(15.6)
	Several times per month or once per month	6	(20.0)	11	(36.7)	11	(36.7)	28	(31.1)
	Never	18	(60.0)	16	(53.3)	14	(46.7)	48	(53.3)
Are you familiar with the Australian Guide to Healthy Eating standard serve sizes?									
	No	16	(53.3)	14	(46.7)	17	(56.7)	47	(52.2)
	Yes^b^	14	(46.7)	16	(53.3)	13	(43.3)	43	(47.8)

^a^height and weight reported for *n* = 29 in Information only group; ^b^‘I have heard about them’ and ‘Yes, I know them’ re-categorised to ‘Yes’; ^c^‘Daily ‘AND ‘Several times per week’ re-categorised to ‘Daily or several times per week’, ‘Once per month or less’ AND ‘Never re-categorised to ‘Once per month or less or Never ‘; ^d^ ‘Daily ‘AND ‘Several times per week’ re-categorised to ‘Daily or several times per week’, ‘Several times per month’ and ‘Once per month’ re-categorised to ‘Several times per month or Once per month’.

### Estimation error

**Fig. 3:**
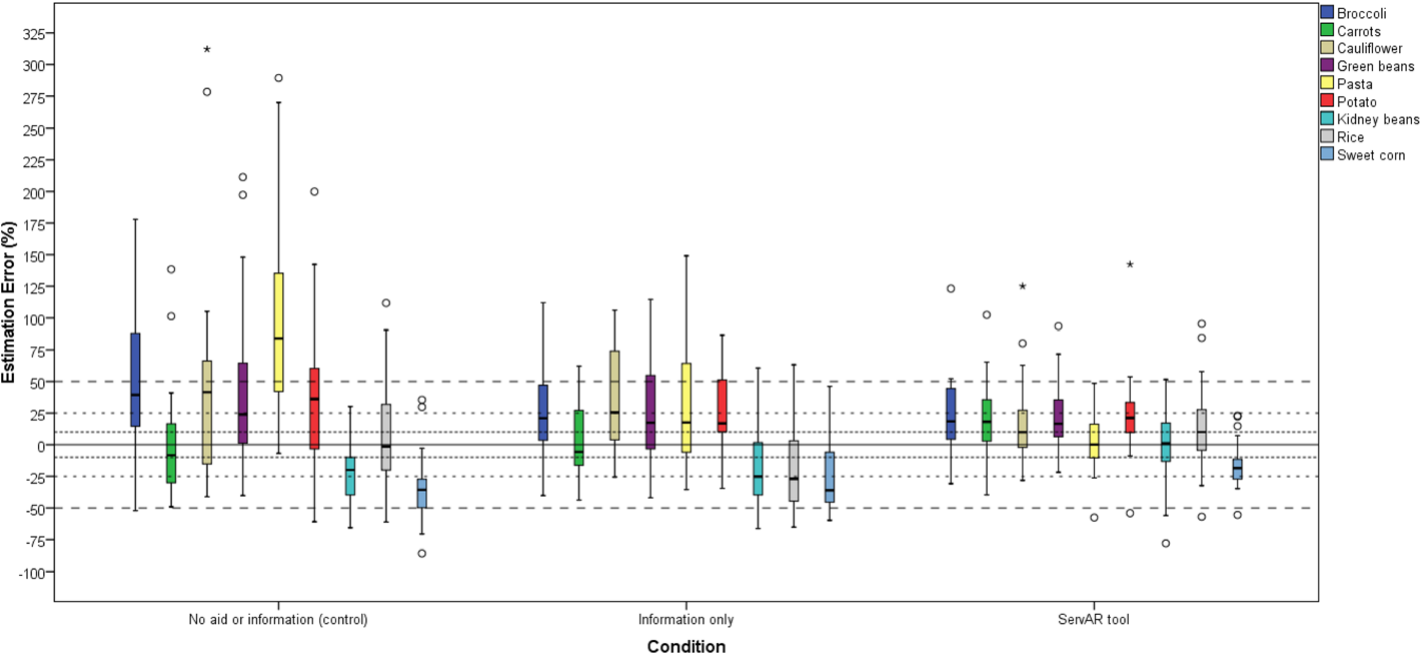
Percentage estimation error for each test food by experimental condition. The distribution of estimation error across conditions is displayed in the form of box-and-whisker plots for each food. The length of each box represents the interquartile range for the estimation error (the outer horizontal boarders represents the 25th and 75th percentiles), and the line drawn across the box represents the median error value. The crossbar of each whisker of the box represents the minimum and maximum error values. Outliers are indicated by open circles (○) and extreme values by stars (⋆). Horizontal lines are used to indicate levels of accuracy within 10% (), ±25% (), and ±50% ()

Table 2 summarises the median food serving weights for each condition. Using the ServAR tool resulted in four of the nine foods being served in a comparable amount to the reference serving, in contrast to the control and information only groups which had two foods and one food, respectively. Servings made with the assistance of the ServAR tool had lower variation compared to those made with information only or no information or serving aid (Fig. 3). For the group using ServAR, the median difference between the amounts served as the reference serve varied between 0.1% for pasta to 21.2% for potato. In contrast, for the control group median errors ranged from −20.0% (kidney beans) to 84.1% (pasta), while for the information only group errors ranged between −26.1% (rice) to 25.5% (cauliflower). Of the total number of servings completed per group (*N* = 270), the ServAR group had a greater proportion within ±10% error (30.7%, *N* = 83) compared to the information only group (19.6%, *N* = 53) and the control group (10.0%, *N* = 27). When the error margin was expanded to ±25%, the cumulative proportion of servings within this range was highest in the ServAR group (65.2%, *N* = 176), followed by the information only (47.4%, *N* = 128) group and the control (33.7%, *N* = 91) group. Finally, 90.7% (*N* = 245) of all servings for the ServAR group were within ±50%, compared to 77.4% (*N* = 209) and 68.9% (*N* = 186) of all servings made by the information only and control groups, respectively.

**Table 2:** Test foods and self-served amounts of test foods by experimental condition

				Real food equivalent self-served amount (g) by condition								
Test Food	Average replica foods in reference serving size^a^ (g)	Convers-ion Factor	Real food equivalent of reference serving size^b^ (g)	Control (*N* = 30)			Information only (*N* = 30)			ServAR tool (*N* = 30)		
				*M*	*IQR*	*P*	*M*	*IQR*	*P*	*M*	*IQR*	*P*
Broccoli	58.5	0.838	49.0	68.3	36.0	<.001*	59.3	21.4	.003*	58.0	19.7	<.001*
Carrots	47.2	1.100	51.9	47.6	24.2	*.688*	49.0	22.6	*.959*	61.3	17.1	.001*
Cauliflower	57.7	0.781	45.1	63.8	36.7	.001*	56.6	31.6	<.001*	49.6	13.3	*.007*
Green beans	56.8	0.730	41.5	51.5	26.3	.001*	48.7	24.1	.003*	48.4	12.0	<.001*
Pasta	54.2	0.834	45.2	83.2	42.1	<.001*	53.2	31.7	.005*	45.2	12.1	*.765*
Potato	81.7	0.907	74.1	100.9	47.2	.001*	86.6	30.4	.001*	89.8	17.7	<.001*
Kidney beans	77.2	1.087	83.9	67.1	25.0	<.001*	62.8	34.8	.003*	84.8	25.5	*.719*
Rice	73.2	1.813	132.7	131.0	68.9	*.453*	97.0	63.5	.001*	145.9	42.6	*.043*
Sweet corn	79.7	1.040	82.9	53.3	18.7	<.001*	53.0	32.8	<.001*	67.6	13.0	<.001*

*M* Median, *IQR* Interquartile range

*Significantly different; Bonferroni corrected *p*-value applied

^a^Average of triplicate weights of replica foods in the reference serving size (1/2 cup)

^b^As validated replica foods were used as the test foods, a real food equivalent weight needed to be calculated. Weight of average replica food serving multiplied by conversion factor to calculate weight of real food equivalent of reference serving size; Wilcoxon signed-rank test compared weight of real food equivalent in reference serving size to median real food equivalent served weight

Rice and sweet corn were reported as the most difficult foods to estimate by the majority of the ServAR group (60.0%, *N* = 18), compared to potato (40.0%, *N* = 12) and rice (30.0%, *N* = 9) in the information only group; and rice and kidney beans (both 30.0%, *N* = 9) in the control group. In contrast, potato (50.0%, *N* = 15) was reported as the easiest to estimate in the ServAR group. Rice and broccoli were reported equally as the easiest foods to estimate in the information only group (both 30.0%, *N* = 9) and in the control group in addition to pasta (all 20.0%, *N* = 6).

### Usability

The mean score for the nine questions relating to the usability of the ServAR tool including potential for regular use and the addition of extra features are summarized in Table 3. Compared to the other two groups, those in the ServAR group had more exposure to the AR tool using it both for the experimental component with the nine foods and the usability component with one food. Therefore, while scores between groups for each item were not significantly different, there are some patterns which may be of interest. Three of the four items rating the existing features of the AR tool had slightly stronger agreement in the ServAR group compared to the other two groups. For the ServAR group, the ability to visualise the served food in the presence of the virtual food overlay was rated with a weaker level of agreement compared to the other two groups. In contrast, agreement on the potential use of the ServAR tool in daily life was similar between groups for two of the three items, with those in the ServAR group in stronger agreement for the potential of the tool to assist people in eating healthier compared to the control and information only groups. Agreement towards the proposed additional features for the ServAR tool was slightly stronger among those in the control group compared to the other two conditions.

**Table 3:** Usability evaluation of the ServAR tool

		Control (*n* = 30)		Information only (*n* = 30)		ServAR tool (*n* = 30)		*ANOVA*	
Questions^a^		*Mean*	*SD*	*Mean*	*SD*	*Mean*	*SD*	*F*_*(2,89)*_	*P*
1. How do you evaluate the following properties of the application:	The app helped me to estimate the size of a standard serve	2.1	1.0	1.8	1.1	1.7	1.0	*1.188*	*.310*
	The app worked well	2.2	1.1	2.3	1.0	2.0	0.9	*.411*	*.664*
	I found it easy to use the app	2.5	1.3	2.4	1.2	2.1	1.1	*.925*	*.400*
	I found it easy to see the real food underneath the portion image overlay	2.3	1.5	2.4	1.4	2.8	1.4	*1.242*	*.294*
2. How do you assess the potential of the application in everyday life:	The app will be helpful to educate consumers about the standard serve sizes	1.7	0.9	1.7	1.0	1.7	1.1	*.011*	*.989*
	The app could help people to control their portion sizes	1.6	0.8	1.6	1.0	1.7	1.0	*.084*	*.919*
	The app could help people to eat healthier	2.1	0.9	2.2	1.4	1.9	1.1	*.366*	*.694*
3. Which features should an app for portion size education have:	It should tell me the energy/cal (kJ/kcal) content of the portion	1.8	1.3	2.0	1.5	2.0	1.4	*.210*	*.811*
	It should adjust with the amount served to tell me the portion size	1.6	0.9	2.1	1.3	2.1	1.3	*1.858*	*.162*

*SD* Standard Deviation

^a^Questions answered on a 6-point Likert scale with ‘1’ = strongly agree to ‘6’ strongly disagree; lower score indicates higher agreement with statement. In the context of these questions, the ServAR tool was referred to as the ‘application’ or ‘app’

## Discussion

This study evaluated the impact of an AR tool, ServAR, on estimation error associated with standard serve serves for nine commonly consumed foods. Use of ServAR improved serving accuracy, with more servings closer to the reference serve compared to control and information only groups. In addition, estimations of standard serve sizes performed with the assistance of ServAR were more consistent and had less variation compared to the other two conditions.

The acceptable level of accuracy associated with portion size estimation error varies depending on the setting. For example, Lucas et al. [30] used an error range of ±10% for estimations undertaken in real-time with the assistance of reference food photographs, while Godwin et al. [31] used a target of within ±20% for accurate recall of food portions and Lee et al. [32] considered estimates within ±15% of the actual food weight to be accurate in the context of automated analysis of image-based dietary records. In the current study, a higher proportion of estimates were within ±10% of the weight of reference serve for ServAR (30.7%) compared to the information only (19.6%) and control (10.0%) conditions. This tendency to perform more accurate estimations with the AR tool continued with almost two-thirds (65.2%) of all estimates made with ServAR within ±25% compared to 47.4% of estimates made with the knowledge of standard serving sizes and 33.7% made without the assistance of an aid or information.

Large variability of individual estimation error is common in studies evaluating the use of aids to assist in the estimation of food portion sizes in real-time [33, 34]. This heterogeneity in the ability to estimate accurately was also demonstrated in the current study. As the presence of an aid is known to improve estimation accuracy, the reduction in median error was expected. However, the improved consistency with which estimations of the standard serve sizes were made by individuals when using the ServAR group is encouraging and further research on its utility in other settings is warranted.

To our knowledge, only two other studies have used an AR tool as an aid for portion size estimation of foods in order to guide individuals in serving and consuming appropriate amounts of foods [21, 22]. In early testing of their AR application, Stutz et al. tested two user input features, both involving touch gestures (one a 3-point gesture and the other a mesh deformation gesture) to estimate the portion size of rice, resulting in errors of 22.7 and 33.7%, respectively [21]. In the experimental condition, the ServAR tool did not require any on-screen user interaction and resulted in an average error of 10.0% for rice. In addition, of interest for this food item in the current study was the result for the control group, which recorded the lowest error amongst the three groups with a difference of −1.3% compared to −26.9% for standard information group. The median estimation error recorded for cooked rice using the ServAR tool is smaller compared to aids consisting of hands or standard household measuring cups [35].

Although use of the ServAR resulted in greater levels of accuracy, it is relevant to note that improved estimation accuracy was also achieved by providing participants with information on standard serving sizes compared to the control group. This finding supports earlier work, which demonstrated that education or training on portion size, including with the use of various aids, is effective in reducing estimation errors [36–40]. In addition, it highlights the potential for information on recommended serve sizes to be incorporated into ServAR to supplement the virtual food portions.

The test foods used in the current study were predominantly amorphous in type and identical in appearance to the virtual overlays. Therefore, evaluation of the ServAR tool using a broader range of foods, including solid and liquids, as well as portion sizes is required. In addition, given the diversity possible in the presentation of the same foods (e.g. carrot presented may be present as batons, rounds or mashed), the application of generic shapes for use within the AR tool warrants investigation. Such aids have been found to be an acceptable alternative to assist in the estimation of wedge-shaped foods [41] and amorphous foods [42] in dietary recall studies, and their usefulness for guiding the serving of food in real-time requires further research.

Internationally, many systems are in place to educate consumers on serving size guidance. However, these systems have mostly been ineffective due to inconsistencies in the presentation of information, unavailability of practical resources, and inherent challenges within the food environment including consumer perceptions and social norms [43]. In addition, using aids to assist with managing portion sizes is seen as inconvenient, time consuming and only needed for those on special diets [14, 44], although usefulness for specific foods, such as grains, is acknowledged [44]. A lack of practical and portable tools is a factor contributing to the low uptake of portion control aids. Scales to weigh foods and measuring jugs were seen as the least convenient, while household measuring cups were seen as most usable [44]. However, these tools also have practical limitations, which may explain why these types of aids are more likely to be used for meals prepared at home, and less likely to be used when eating out or for special occasions [44]. Further, serving size guidance must be viewed as relevant to one’s eating behaviours [14]. ServAR offers a portable and convenient solution to assist individuals to manage portion sizes.

ServAR was reported by participants as a useful aid to guide the serving of food, to educate on standard serving sizes and to support portion control as part of healthy eating behaviours. For participants that also used the AR tool to estimate all nine foods, in addition to the usability activity, high agreement for overall ease of use and assistance with estimating the standard serve size indicate that satisfaction may increase with extended use. Compared to the other two groups, those in the ServAR group had more exposure to the AR tool using it both for the experimental component and the usability activity in which all participants manipulated the position of the iPad Mini to align the virtual and real fiducial markers. This distinction is important in interpreting the findings as those in the ServAR group had some difficulty viewing the real food underneath the virtual overlay. In addition to the level of opacity of the virtual foods, the contrast of the white-coloured foods against the plate made estimation challenging for some individuals. Further, aligning the virtual fiducial marker with the fiducial marker present in reality was also found to be challenging, particularly when combined with the task of serving food. These factors highlight areas of the ServAR tool that should be refined before further use.

Similar interaction challenges were noted by Stutz et al. and Domhardt et al. in the testing of their AR applications [21, 22]. For example, participants found it difficult to manipulate the position of the device with reference to the fiducial marker in their AR application for estimation of food portions [22]. In this study six participants were required to perform an AR task to outline food portions in order to estimate portion size, with this task reported as being too complex and/or too imprecise by half of participants [22].

There are two important limitations in the current study that must be considered when interpreting the results. First, the ServAR was tested on nine foods, with eight of these amorphous, and all foods compared to one portion, therefore limiting the generalisability at this point. Evaluations with a broader range of foods, including energy-dense, nutrient poor foods (e.g. chips or chocolates) that incorporate an expanded number of virtual serving guides and which are undertaken in both controlled (i.e. laboratory-based) and free-living settings are needed before the true utility of ServAR can be determined. Secondly, the accuracy of the ServAR tool for estimating varying portion sizes of the same foods needs to be established, as variations exist in the perceptions along the continuum of portion size from ‘small’ to ‘large’ [45]. However, this was the first study to systematically test an AR tool requiring minimal on-screen user interaction is comparison to standard serving sizes in a large sample.

## Conclusion

Use of the AR tool improved serving accuracy and consistency among users. ServAR demonstrates potential as a practical tool to support the accurate serving of food for portion control. Further evaluation across a broad range of foods, portion sizes and settings is warranted.
